# The Atlanta Medical College

**Published:** 1872-09

**Authors:** 


### THE ATLANTA MEDICAL COLLEGE.

In the last number of THE COMPANION an advertisement of this College appeared in our advertising department. This no doubt astonished our readers—as it did the editors—when the position of this journal was so well known in reference to it. We "rise to explain" that it was placed therein without our knowledge, consent or approval. We cannot consciencciously give that endorsement of the institution which our advertising department would seem to secure for it. This much common honesty and manliness on our part requires us to say, under the circumstances.

